# The Effect of Surface Functionalization of Magnesium Alloy on Degradability, Bioactivity, Cytotoxicity, and Antibiofilm Activity

**DOI:** 10.3390/jfb16010022

**Published:** 2025-01-12

**Authors:** Morena Nocchetti, Michela Piccinini, Donatella Pietrella, Cinzia Antognelli, Maurizio Ricci, Alessandro Di Michele, Layla Jalaoui, Valeria Ambrogi

**Affiliations:** 1 Department of Pharmaceutical Science, University of Perugia, 06123 Perugia, Italy; morena.nocchetti@unipg.it (M.N.); michela.piccinini@dottorandi.unipg.it (M.P.); maurizio.ricci@unipg.it (M.R.); layla.jalaoui@libero.it (L.J.); 2 Department of Medicine and Surgery, University of Perugia, 06132 Perugia, Italy; donatella.pietrella@unipg.it (D.P.); cinzia.antognelli@unipg.it (C.A.); 3 Department of Physics and Geology, University of Perugia, 06123 Perugia, Italy; alessandro.dimichele@unipg.it

**Keywords:** magnesium alloy, bone implants, surface functionalization, stability, in vitro bioactivity, cytotoxicity, antibiofilm activity

## Abstract

Magnesium alloys are promising biomaterials to be used as temporary implants due to their biocompatibility and biodegradability. The main limitation in the use of these alloys is their rapid biodegradation. Moreover, the risk of microbial infections, often following the implant surgery and hard to eradicate, is another challenge. Thus, with the aim of reducing biodegradability and conferring antibiofilm activity, sheets of the magnesium alloy AZ31 were properly modified with the introduction of hydroxy (polyethyleneoxy)propyl silane (PEG) and quaternary ammonium silane chains (QAS). The derivatized sheets were characterized by ATR-FTIR spectroscopy and their performances as concerns their stability, Mg^2+^ in vitro release, and in vitro bioactivity were evaluated as well. The results showed an increased stability with a reduction in corrosion, a slower Mg^2+^ ion release, and the formation of hydroxyapatite in the sheets’ surface. In addition, cytotoxicity evaluations were carried out on human gingival fibroblasts showing that the AZ31 and AZ31-PEG plates had good cytocompatibility. Finally, the antibiofilm activity on *Staphylococcus aureus, Staphylococcus epidermidis*, and *Pseudomonas aeruginosa* was carried out by evaluating the capacity of inhibition of biofilm adhesion and formation. The results demonstrated a significant reduction in biofilm formation by *Staphylococcus epidermidis* on AZ31-QAS.

## 1. Introduction

In the biomedical field, Mg alloys have recently attracted attention as promising materials for temporary and short-term implants due to their biocompatibility, mechanical properties, and biodegradability [1,2]. For applications in orthopedics, important properties are also excellent osseointegration and a low elastic modulus, similar to those of human bone, ideal for preventing the negative effect of stress shielding [3]. Moreover, Mg^2+^ ions are essential for many metabolic pathways, being enzymatic cofactors [4], and, when resulting from implant degradation, can be an aid for tissue healing and growth. The demand for suitable new materials for bone implants is growing, due to the increase in bone fractures caused by diseases, accidents, and aging that very often need surgery. Today, the main osteosynthesis systems are Ti plates and bi-cortical screws, which are not optimal for rib fixation as they can cause further rib fracture [5]. Moreover, all these techniques involve serious complications, and the implants need to be removed through a second surgery. A biodegradable material, such as a Mg alloy, which dissolves in the human body, has been suggested as a valid alternative to permanent metal implants, with benefits for the patient and public health care in terms of costs [3]. However, Mg alloys undergo a rapid degradation in the biological environment, which implies a loss of mechanical properties, thus compromising the implant functionality before the newly formed bone can take on the necessary mechanical load. Concomitantly, the process of rapid biodegradation causes the generation of hydrogen, which can provoke undesirable effects. Hydrogen gas bubbles and alkalization resulting from the fast corrosion of Mg represent further drawbacks that may cause tissue necrosis [3].

Among magnesium alloys, our attention was pointed at AZ31, which has a low Al content, good mechanical properties such as increased strength and plasticity, and acceptable corrosion resistance due to the presence of Al. Recently, AZ31 has been proposed for the manufacturing of rib fixators employed to help the osteosynthesis of multifractured ribs [6,7,8].

It is known that surface modifications of Mg alloys can increase their resistance to corrosion and a coating can considerably improve the degradation times and, therefore, its biocompatibility. Thus, Mg alloys are submitted to surface treatment, which consists of forming a protective layer that hinders contact between Mg alloys and the physiological environment. Among the proposed techniques, Mg alloys have been coated with Mg-Al layered double hydroxide [9], plasma electrolytic oxidation, which fabricates a porous ceramic coating and is followed by sealing pores by polycaprolactone [10], or poly(L-lactide) [11], poly(lactic-co-glycolic) acid [8,12,13,14], calcium phosphate [15], hydroxyapatite [16,17], and organosilanes [18,19,20,21,22].

Organosilanes are bifunctional reagents with the general structure (R_0_O)_3_Si–R–X. Where R is an alkyl chain, R_0_ can be Cl, F, CH_3_, or C_2_H_5_ or other substituents and X is a functional group such as a thiol, amine, carboxylic acid, alcohol, or alkyl group. The alkoxysilane portion of organosilanes is able to bond with metal surfaces through complex hydrolysis/condensation reactions with the formation of Si–O–metal bonds [22,23]. As is known, the occurrence of fractures is always accompanied by inflammation and bacterial infections [24]. Staphylococci, which are often responsible for infections, can grow within a biofilm, which protects them from host defenses, antibiotic therapies, and biocides. Therefore, besides the above-described chemical requirements, it is also important that Mg alloys are endowed with antibiofilm activity. Indeed, the pH created following the degradation of the alloy makes the environment inhospitable to microorganisms, and bacteria do not adhere to the areas where corrosion occurs. However, following the slowing down of corrosion, the pH of the surrounding environment becomes less alkaline and therefore less inhospitable for any microorganisms. Thus, providing a coating able to reduce the corrosion rate and to give antibacterial properties is a mandatory challenge.

In this paper, surface modifications of the AZ31 Mg-based alloy were performed in order to improve its performance as concerns the biodegradability times and antibiofilm properties. AZ31 was functionalized by pegylation and by adding a quaternary ammonium salt and the functionalized sheets were evaluated for their in vitro biodegradability in simulated body fluid (SBF) and antibiofilm, as well as cytotoxicity activities.

## 2. Materials and Methods

### 2.1. Materials

Magnesium alloy AZ31, whose composition was Zn 1%, Al 3%, Mg 96%, was purchased from GoodFellow (Hamburg, Germany). Dimethyloctadecyl(3-trimethoxysilylpropyl)ammonium chloride (QAS) and hydroxy(polyethylyenoxy) propyl] triethoxysilane (8-12EO) 50% in ethanol (PEG-silane) were purchased from Zentek srl, (Milan, Italy). Sodium chloride, magnesium chloride hexahydrate, sodium bicarbonate, dibasic potassium phosphate trihydrate, potassium chloride, hydrochloric acid, calcium chloride, sodium sulfate and tris(hydroxymethyl)aminomethane, methanol, and ethanol were purchased from Sigma-Aldrich (Milan, Italy). Sodium hydroxide and nitric acid were purchased from J.B Baker (Segrate, Milan, Italy). Ultrapure water (ρ = 18.3 MΩ×cm@25 °C) was obtained from Synergy^®^ UV Water Purification System (Millipore Sigma, Rome, Italy). All other chemicals and solvents were analytical grade and were used as received.

### 2.2. AZ31 Attivation

The AZ31 samples (0.25 mm thickness) were polished with SiC emery papers from 400 up to 4000 grit and washed in an ultrasound bath, firstly in acetone, then in ethanol, and finally in deionized water for 10 min. After cleaning, samples were air-dried [25]. These samples were soaked in a 3 M sodium hydroxide solution and heated at 75 °C for 90 min. Then, samples were rinsed with ultrapure water and allowed to dry at room temperature. Lastly, they were heated for 30 min at 80 °C and then for another 30 min at 120 °C. Samples were maintained in the oven while the oven temperature increased from 80 to 120 °C [14].

### 2.3. AZ31 Derivatization with QAS

Firstly, a solution (5 mL) composed of 4.36 mL of methanol, 0.34 mL of QAS, 0.3 mL of deionized water, and one drop of NaOH 3 M was prepared and allowed to stay at room temperature for 1 h to obtain hydrolysis of QAS. Then, activated AZ31 samples were vertically soaked in this solution for 1 h and then were washed with deionized water and finally air-dried [20]. This sample will be hereinafter noted as AZ31-QAS.

### 2.4. AZ31 Derivatization with PEG-Silane

Firstly, a solution (5 mL) composed of 4 mL of ethanol, 0.70 mL of PEG-silane solution, 0.3 mL of deionized water, and one drop of NaOH 3 M was prepared and allowed to stay at room temperature for 1 h to obtain hydrolysis of PEG-silane. Then, the activated AZ31 sheet was vertically soaked in this solution, previously heated at 40 °C, for 2 h. Finally, the sheet was washed with deionized water and was air-dried. This sample will be hereinafter noted as AZ31-PEG.

### 2.5. Characterization

The X-ray diffraction (XRD) patterns were recorded with a Panalytical X’PERT PRO MPD diffractometer operating at 40 kV and 40 mA, with a step of 0.03° and a step time of 150 s using Cu Kα radiation and an X’Celerator detector (PANalytical, Royston, UK). The Bruker DIFFRAC.EVA V5 software equipped with COD database was used for the phase identification.

Attenuated total reflectance FTIR (ATR-FTIR) spectra were recorded using an FTIR Shimadzu IR-8000 spectrophotometer (Shimadzu, Europa GmbH, Duisburg, Germany) equipped with a total attenuated reflectance FTIR spectrum acquisition device. The spectral range collected was 400 to 4000 cm^−1^ with a spectral resolution of 4 cm^−1^ acquiring 100 scans.

Sample morphology was evaluated using Scanning Electron Microscopy (SEM) by placing the sample on an aluminium stub previously covered with a graphite conductive adhesive and metalized with chromium (a 5 nm layer) for 20 s. An FEG LEO 1525 ZEISS instrument (Oberkochen, Germany) was used. The element mapping images were performed using an EDX (Energy-dispersive X-ray spectroscopy) analyzer (Bruker Quantax) (Milano, Italy) coupled to the electron microscope.

### 2.6. In Vitro Degradation in SBF

In vitro degradation tests were carried out with 8 × 4 mm sheets in 10 mL of simulated body fluid (SBF) [26] at 37 °C in static conditions. The sheets were previously sealed on one side with a cyanoacrylic glue, weighted (W1), and soaked in the fluid by placing them at the bottom of a flask with the free side facing the fluid. At predetermined time intervals (1, 7, 15, 21, and 30 days), the sheets were recovered, washed with deionized water, dabbed with a filter paper to remove the excess water, and finally weighted again (Wt). Every week, the fluid was renewed. The percentage of weight loss was determined with the following formula:

$$\%W.L.=\left(W1-\frac{Wt}{W1}\right)\times 100$$

W1 = initial weight;

Wt = weight at time t.

During the test, the pH of the fluid was measured by a pH meter (pH 211 microprocessor pH-meter, Hanna Instruments, Woonsocket, RI, USA). The experiment was performed three times, and the values are reported as the average of three determinations ± standard deviation (S.D.).

### 2.7. Release of Mg^2+^ Ions from AZ31 Sheets in SBF

AZ31 sheets (8 × 4 mm) were immersed into 10 mL of SBF at 37 °C in static conditions. At predetermined time intervals, a quote of fluid was collected and compensated with the same amount of the fresh SBF. The Mg^2+^ concentration in each withdrawal was determined after 1 mL conc. HNO_3_ addition and proper dilution by inductively coupled plasma emission spectrometer (ICP-OES) (Varian 700-ES series, Agilent Technologies, Mulgrave, Victoria, Australia).

### 2.8. In Vitro Bioactivity Test in SBF

In vitro bioactivity of the sheets (8 × 4 mm) was evaluated according to Kokubo’s method [26]. Samples were immersed in SBF and maintained at 37 °C for 30 days. The SBF fluid was changed once a week. The samples were recovered after 1, 7, 15, and 30 days, washed with deionized water, P_2_O_5_-dried, and monitored by SEM, EDX, ATR-FTIR, and XRD.

### 2.9. Cell Culture

BSCL138 human gingival fibroblasts (IZSLER, Brescia, Italy) [14,27] were cultured in Eagle’s minimum essential medium (MEM, Thermo Fisher Scientific, Waltham, MA, USA) supplemented with 10% fetal bovine serum (FBS, Thermo Fisher Scientific, Waltham, MA, USA), penicillin (10,000 U/mL), streptomycin (10,000 μg/mL), and 25 μg/mL amphotericin B as anti-fungal agent (Thermo Fisher Scientific, Waltham, MA, USA) [27]. Briefly, cells were incubated at 37 °C in a humidified 5% CO_2_ atmosphere. Upon 80% confluence, cells were detached with trypsin 0.25% in EDTA (Gibco, Paisley, UK). After 10 min of trypsinization, complete medium was added to inactivate trypsin. The cells were centrifuged for 10 min at 800× *g*. Cell pellets were re-seeded in apposite plates and used for cell viability assay.

### 2.10. Cell Viability Assay

Cell viability was performed by 3-(4,5-dimethylthiazol-2-yl)-2,5-diphenyl-tetrazolium bromide (MTT) assay (Sigma Chemical Co., St. Louis, MO, USA), as previously described [28]. Briefly, cells were seeded at the concentration of 3 × 10^4^ cells/well in 24-well plates (Euroclone, Pero, MI, Italy) with 500 µL of DMEM and incubated at 37 °C in a humidified 5%, CO_2_ atmosphere. After 24 h, cells were treated with the sheets (disk of 5 mm diameter) for 24 h. The untreated cells (control) received only fresh medium. At the end of the treatment period, 1×MTT reagent (stock solution 5 mg/mL) was added to each well and the plate was incubated at 37 °C for 4 h. The formazan crystals were dissolved in 0.01 N HCl/10% SDS solution at 37 °C in a humidified 5%, CO_2_ atmosphere overnight. After that, the absorbance of each well was evaluated spectrophotometrically at 550 nm. The amount of color produced was directly proportional to the number of viable cells. Data represent the means ± SD of two independent experiments each performed in triplicate. One-way analysis of variance (ANOVA) was performed. *p*-values of <0.05 were considered significant.

### 2.11. Microorganisms

The microbial strains used in this study were *Staphylococcus aureus* (ATCC 29213), *Staphylococcus epidermidis* (ATCC 12228) as two Gram-positive bacteria and the Gram-negative *Pseudomonas aeruginosa* (ATCC 15692). The stock cultures were maintained at −20 °C. After recovery, bacterial strains were maintained in Muller Hinton Agar (MHA). The day before the test, one colony was inoculated in the appropriate culture medium (Muller Hinton Broth, MHB) and incubated for 24 h at 37 °C.

### 2.12. Adhesion and Antibiofilm Activity

To prepare the bacterial suspension, a single colony of the stock culture was streaked onto MHB and incubated at 37 °C for 24 h. Cells of the resultant culture were harvested, washed twice with sterile phosphate-buffered saline (PBS) (pH 7.2) at 2000× *g* for 10 min, and resuspended in MHB plus 2% sucrose.

The adhesion and biofilm formation assays were carried out following Wady A.F. et al. [29] with some modifications. All sheets (disk of 5 mm diameter), after sterilization under UV light, were placed on the bottom of a 96-well culture microplate. Then, 200 μL of the different bacterial suspension (10^5^ cells/mL) was added.

For the adhesion test, cells were incubated for 90 min.; for the biofilm assay, for 24 h at 37 °C. After incubation, non-adherent bacteria were removed from the disks by gently washing twice with sterile saline. Each sample was placed in a tube containing 1 mL of sterile saline solution. All tubes were then sonicated in an ultrasonic bath cleaner operating at 47 kHz, 234 W for 6 min to detach all bacteria adherent to the disk surfaces, bringing them in suspension. Then, 50 μL of each suspension was serial diluted 1:10 in saline and plated on MHA plates. They were incubated at 37 °C for 24 h and finally CFUs were counted and expressed as CFU/disk. All experiments were carried out in triplicate.

## 3. Results and Discussion

### 3.1. AZ31 Derivatization and Characterization

**Figure 1:**
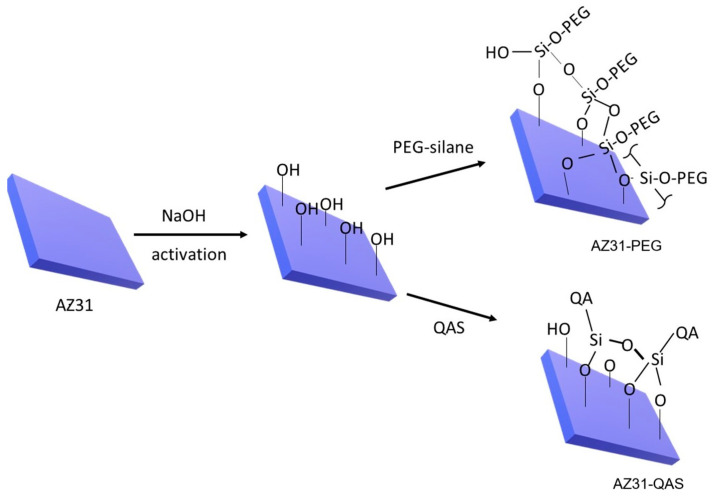
Schematic representation of AZ31 derivatization.

A schematic view of the derivatization of AZ31 described in this research is shown in Figure 1.

**Figure 2:**
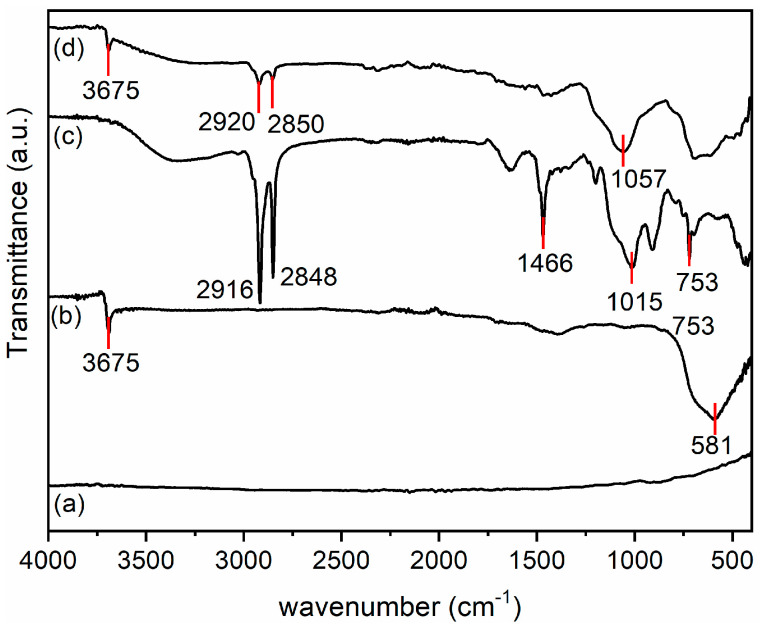
ATR-FTIR spectra of AZ31 (**a**), AZ31-OH (**b**), AZ31-QAS (**c**), and AZ31-PEG (**d**).

The first step for the derivatization of the magnesium plate consisted of its activation by treatment with NaOH as described above [20,25]. The successful activation was confirmed by the ATR-FTIR spectrum (Figure 2). In fact, the non-activated AZ31 alloy did not show any absorption on its surface, whereas, after treatment with NaOH, the absorption peak at about 3675 cm^−1^, attributable to the stretching of the –OH groups formed on the plate surface after activation [25] and the band at 581 cm^−1^ due to the presence of MgO [30], could be detected. Subsequently, derivatization was performed by pegylation with PEG-silane and by the introduction of a quaternary ammonium function with QAS, following the previously described procedure properly modified [20]. The derivatization of the AZ31 sheets was confirmed by ATR-FTIR. The spectra of the obtained samples are shown in Figure 2. The spectrum of the AZ31-QAS showed a broad band in the region between 3000 and 3590 cm^−1^ due to the –OH stretching of the silanol groups, formed from the hydrolysis of alkoxysilane, or of traces of moisture involved in hydrogen bonds, stretching vibrations of CH_2_ at 2916 cm^−1^ and 2848 cm^−1^ [31,32], bending of CH_2_ at 1466 cm^−1^, typical of the long aliphatic chain of QAS, and, finally, Si-O stretching at 1015 cm^−1^ and its bending at 753 cm^−1^ [32,33]. The spectrum of AZ31-PEG was characterized by the vibrations of pegylated chains with a narrow peak at about 3675 cm^−1^ corresponding to the OH terminal of the chain (or unreacted -OH of the sheets’ surface), with anti-symmetric and symmetric CH_2_ stretching vibrations at 2920 and 2850 cm^−1^, respectively, in analogy with those of AZ31-QAS, but with less intensity. Finally, a broad band around 1057 cm^−1^, which corresponds to asymmetric Si-O stretching [34,35] and C-O stretching of the PEG chain [36], was observed.

The success of the functionalization was also confirmed by SEM and EDX analysis of the AZ31-QAS and AZ31-PEG sheets. The elemental mapping (Figure S1) shows that the surface is uniformly covered by carbon derived from the organic derivative attached to AZ31, with magnesium only visible in a few zones. Note that more efficient coverage is obtained in the AZ31-PEG sample.

After functionalization, the behavior of the sheets in SBF was evaluated over time as concerns their degradation, Mg^2+^ release, changes in pH, and in vitro bioactivity. Before performing these tests, a cyanoacrylic glue, used in surgery, was applied to one of the two sides of the sheet to mimic the conditions of application of the alloy as a rib fixator at the fracture site [6,7,8].

### 3.2. Stability Tests

**Figure 3:**
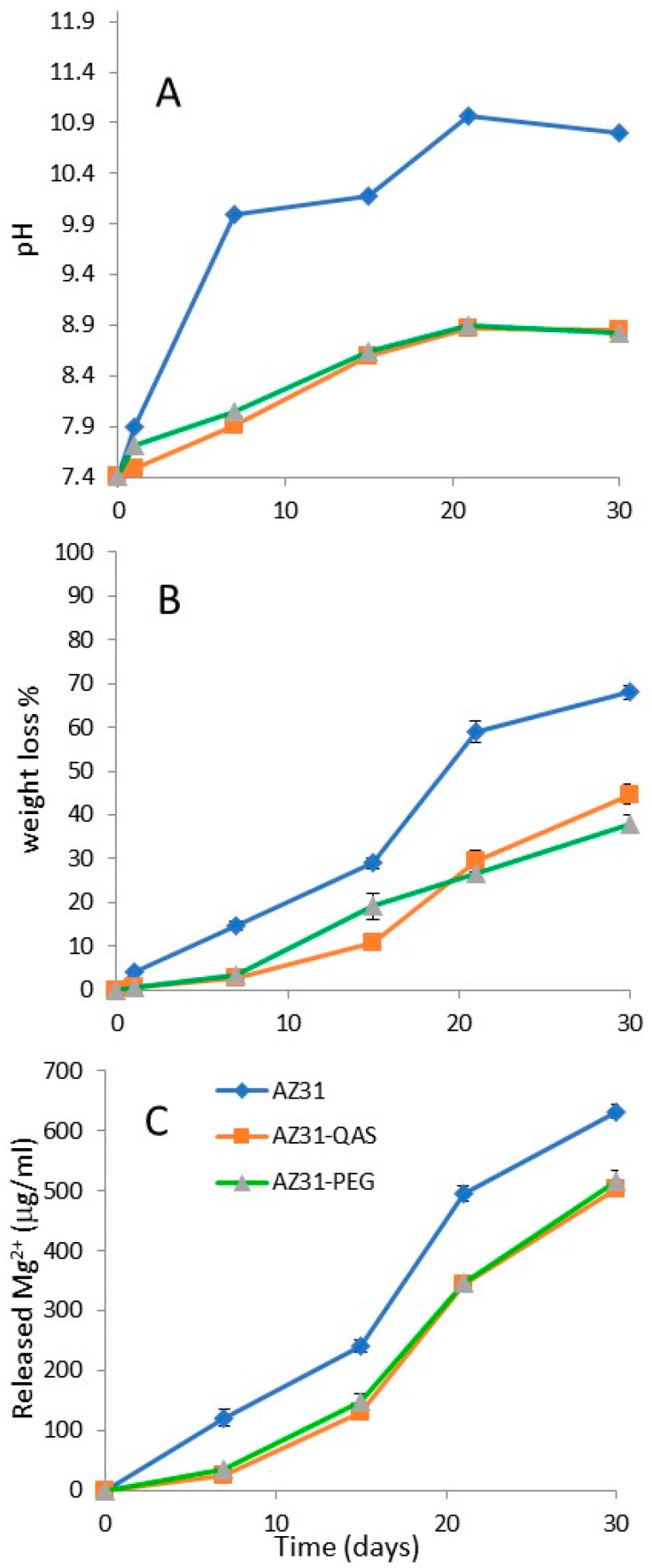
Weight loss (%) of AZ31, AZ31-PEG, and AZ31-QAS (**A**), pH values in SBF for AZ31, AZ31-PEG, and AZ31-QAS (**B**), and Mg^2+^ release from AZ31, AZ31-PEG, and AZ31-QAS in SBF at 37 °C (**C**).

This test was conducted by immersing the slab in SBF at 37 °C and monitoring the weight loss, the pH changes in the fluid, and the Mg^2+^ release. Weight loss (%) was measured after 1, 7, 15, 21, and 30 days, as weight loss was expected after corrosion [37]. Indeed, as shown in Figure 3A, weight loss gradually increased and the loss was greater for the AZ31 sample, whose weight decreased by more than 60% on the 30th day, while the derivatives (AZ31-QAS and AZ31-PEG) underwent a minor weight loss with a maximum value of 35–45% on the 30th day. It can also be observed that there was not a substantial difference between the two derivatized sheets.

These results suggest that derivatization with siloxane markedly improves AZ31 stability [38]. Indeed, when in contact with water, hydrolysis of silanes occurred with the formation of silanol groups (SiOH), which react to the hydrated metal surfaces (metal–OH) via the formation of Si–O–metal bonds [39]. The silanol groups undergo self-crosslinking via siloxane bonds (Si–O–Si) and this leads to an organic protective layer chemically bound to the metallic sheet [40]. The coating protects the sheet from degradation. During the previous test, the pH of the immersion fluid was monitored, and the results (Figure 3B) showed that the pH value of all solutions increased during the immersion time, reaching alkaline pH values. The fluid in which the uncoated AZ31 sample is immersed reaches the most alkaline value (pH almost 10 after 7 days), whereas the derivatized sheets reached pH values of 8.5 after 21 days. This can be explained by the fact that the coating determines the protection and slows down corrosion [25,33]. Figure 3C shows Mg^2+^ release from slabs in SBF as a function of time. The release of Mg^2+^ from AZ31 increased over time and the value of 120 μg/mL was reached at 7 days, indicating that the corrosion resistance of the unmodified magnesium alloy AZ31 was poor, and it degraded rapidly in the human body. The sheet coating improves the alloy resistance to corrosion and, in fact, the concentration of Mg^2+^ released from the two derivatives (AZ31-QAS and AZ31-PEG) at 7 days was 25.9 μg/mL and 34.4 μg/mL, respectively, values significantly much lower than those from the unmodified AZ31 alloy. Even after 30 days of immersion, the amount of Mg^2+^ released from the unmodified plate was greater than that of the derivatives (632 µg/mL versus approximately 502 µg/mL for AZ31-QAS and 516 µg/mL for AZ31-PEG).

### 3.3. In Vitro Bioactivity

**Figure 4:**
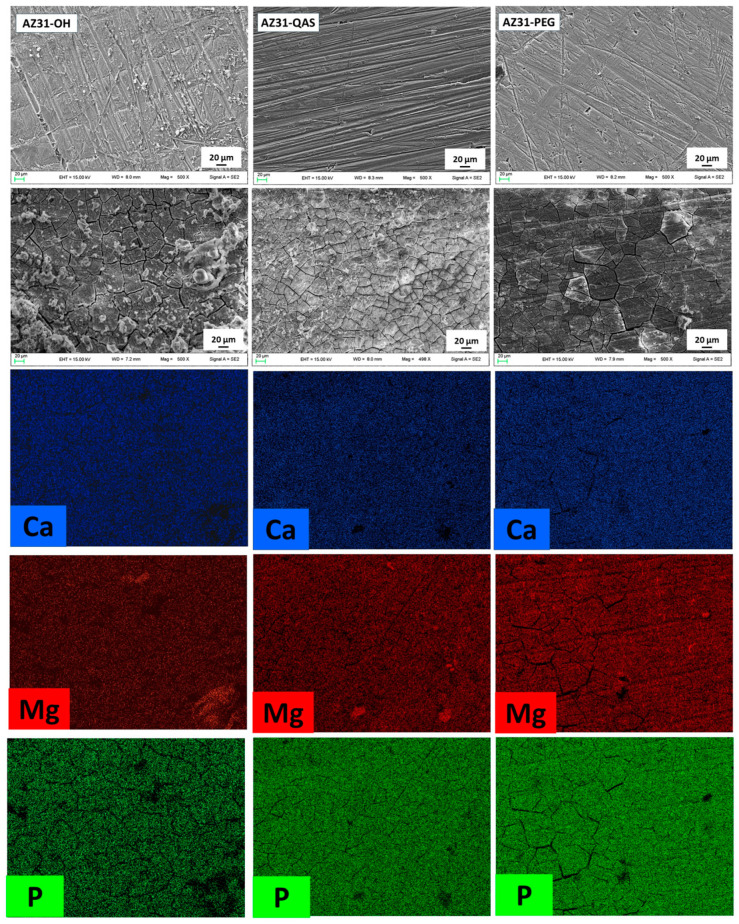
SEM micrographs of AZ31-OH, AZ31-QAS, and AZ31-PEG before (first row) and after 15 days of immersion in SBF (second row) and relative mapping of the shown elements after 15 days.

The bioactivity properties of the biomaterial for bone regeneration are evaluated in vitro by XRD, ATR-FTIR, and SEM by monitoring the eventual hydroxyapatite deposition on the sheet surface following immersion in SBF. The sheets were immersed in SBF, and their surface was monitored by SEM after 1, 7, 15, and 30 days. Micrographs before immersion and after 15 days’ immersion are shown in Figure 4.

For all the samples, evident signs of corrosion, such as cracks, can be observed after 15 days according to a certain weight loss of the sheets recorded in the stability tests. In addition to corrosion signs, for all the samples after 15 days of immersion, the formation of crystals can be observed. The elemental mapping, unlike that obtained before immersion, shows the presence of phosphorus, calcium, and oxygen distributed quite evenly. This could be due to the formation of hydroxyapatite, in agreement with what is reported in the literature [41] regarding the formation of hydroxyapatite on the surface of AZ31. The mapping and EDX spectra after 30 days are shown in Figure S2. The photographs show large corrosion cracks, and the hydroxyapatite layer coverage appears to be different for the three sheets. In particular, AZ31-OH and AZ31-QAS show a fairly uniform distribution of phosphorus and calcium elements, with a few zones of bright spots of magnesium representing uncovered or less covered zones. At the same time, the EDX spectra (Figure S2) show an increase in the presence of phosphorus and calcium. Conversely, elemental mapping of AZ31-PEG shows less hydroxyapatite coverage and the EDX spectrum (Figure S2) shows a moderate increase in phosphorus compared to the other samples, while chloride salts may be present.

**Figure 5:**
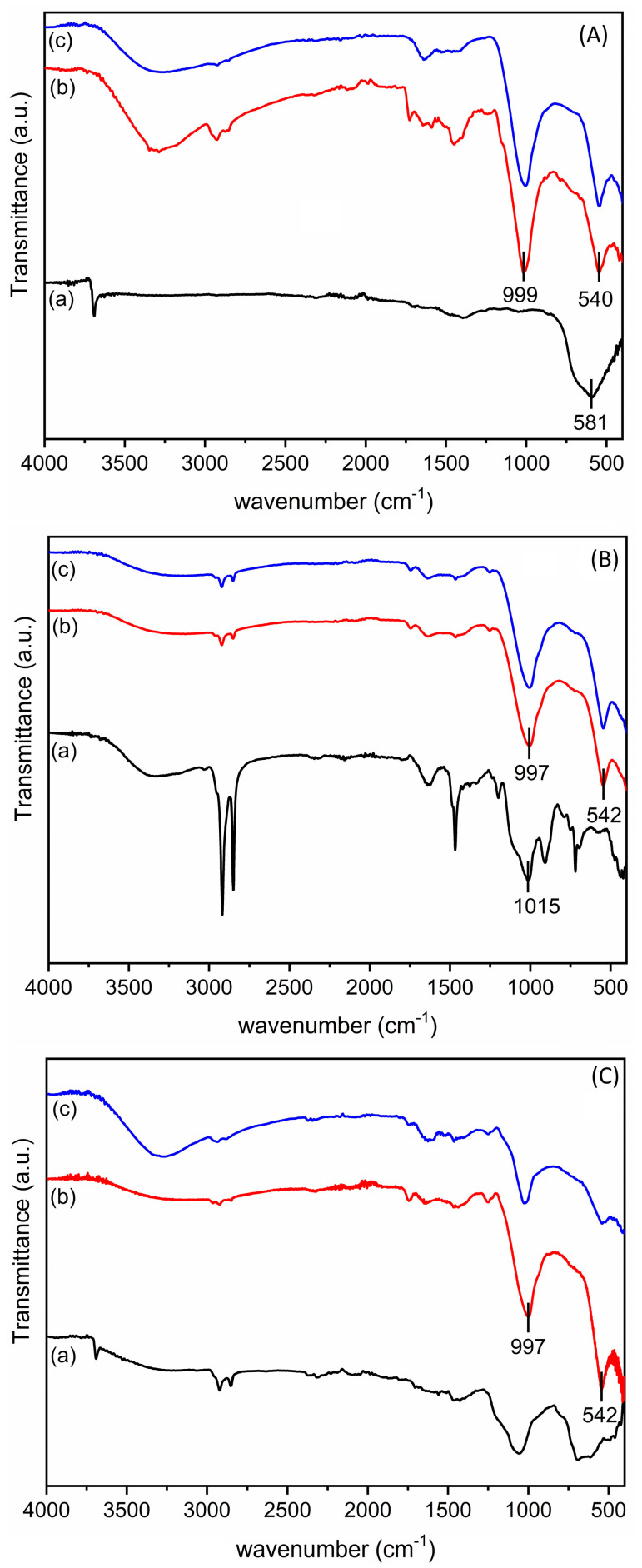
ATR-FTIR of AZ31-OH (**A**), AZ31-QAS (**B**), and AZ31-PEG (**C**) before (a) and after immersion in SBF for 15 (b) and 30 (c) days.

The surface modifications after immersion in SBF were also evaluated, using ATR-FTIR, as hydroxyapatite absorbs between 1000 and 1100 cm^−1^ due to the v_3_ vibration mode of the phosphate group P-O bonds and around 560 cm^−1^ due to the same vibrational motions [42,43]. In Figure 5, the ATR-FTIR spectra of the sheets recovered from SBF were compared with those of the untreated sheet. In all the samples, after 15 and 30 days of immersion in SBF, absorption peaks around 1000 and 540 cm^−1^ appear, and they were assigned to precipitated hydroxyapatite. However, in these samples, it is observed that the absorption band at 540 cm^−1^, related to the bending of the phosphates, overlaps with the absorption of MgO [30]. This suggests that the band at 540 cm^−1^ could be attributable to the presence of both corrosion products and phosphate salts [44]. As for the ATR-FTIR spectra of AZ31-QAS and AZ31-PEG at increasing immersion times, it can be observed that the signals at 2916 cm^−1^ and 2848 cm^−1^ of the CH_2_ stretching [31,32] decrease in intensity. The hydroxyapatite phosphate signal at 997 cm^−1^ overlaps with the Si-O-Si signal at 1015 cm^−1^ typical of QAS and PEG [44] (Figure 5B,C).

**Figure 6:**
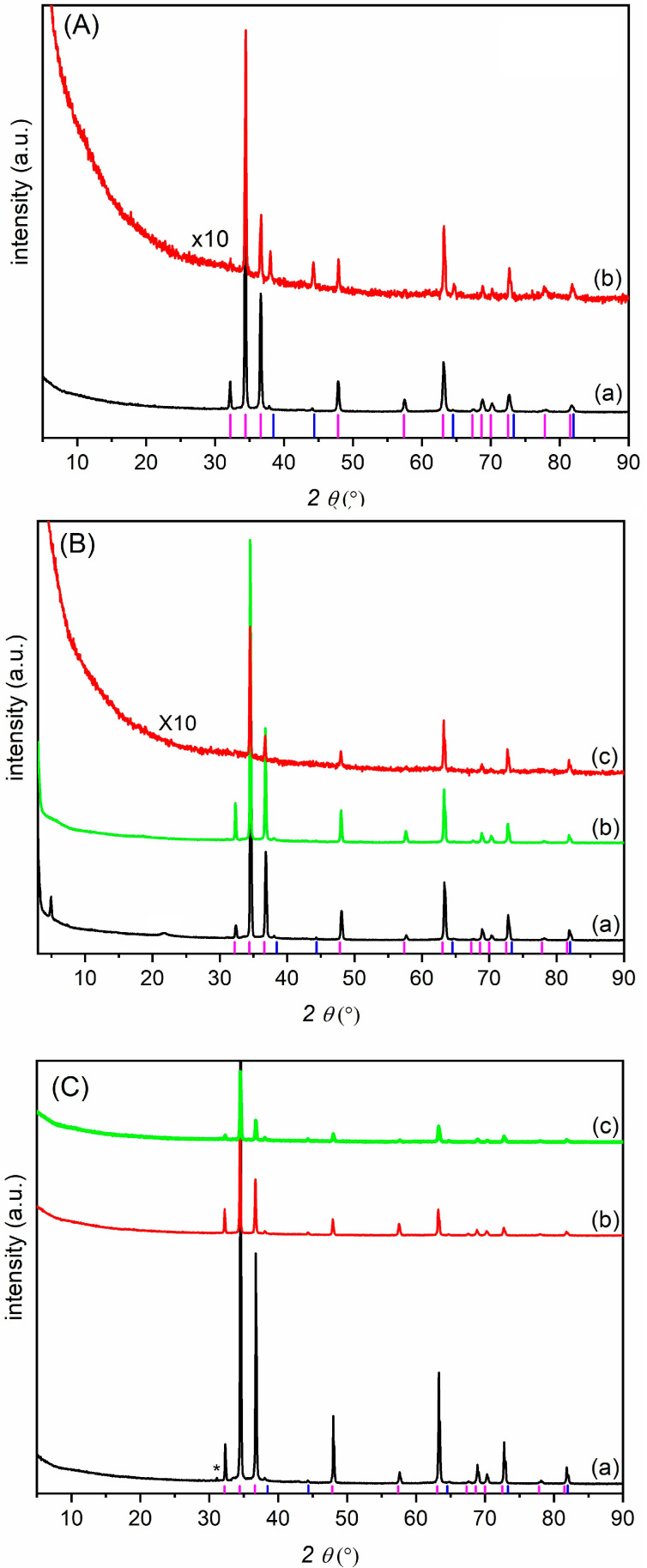
Diffractogram of AZ31 (**A**), AZ31-QAS (**B**), and AZ31-PEG (**C**) as prepared (a), after 15 (b), and 30 (c) days. Blue line: MgO phase (COD1011173); purple line: Mg hexagonal phase (COD1512519); * MgSiO_4_ (COD9000270).

Finally, the samples were also analyzed by X-ray diffractometry. Figure 6 shows the diffractograms of the samples after immersion in SBF and, for comparison, those of the starting samples (AZ31, AZ31-QAS, and AZ31-PEG). In the diffractogram of AZ31 evaluated before and after the bioactivity tests (Figure 6A), two phases are observed, magnesium and traces of magnesium oxide, in agreement with the literature [4,22]. After 30 days of immersion, all peak intensities decreased, and this is a sign that corrosion has occurred or that the pristine phases are covered by a layer of amorphous phases. In this sample, the ratio of MgO/Mg reflection intensities increased, indicating a partial conversion of magnesium to MgO. The diffractograms of AZ31-QAS and of AZ31-PEG show a decrease in the intensity of the reflections after immersion in SBF, very likely due to the deposition of amorphous hydroxyapatite. It is noteworthy that the amount of MgO remains unchanged, demonstrating the anticorrosive effect of derivatization. Furthermore, traces of magnesium silicate (Mg_2_SiO_4_) are observed as a consequence of the reaction of the magnesium surface with the silane groups. In the pattern of the starting AZ31-QAS, a reflection at a low angle was observed, which was attributed to a regular organization of the QAS alkyl chains.

### 3.4. Biological Activity: Cytotoxicity

The cytotoxicity of the magnesium alloy AZ31 and its derivatives (AZ31-QAS and AZ31-PEG) was evaluated using human gingival fibroblasts and by performing the MTT assay.

**Figure 7:**
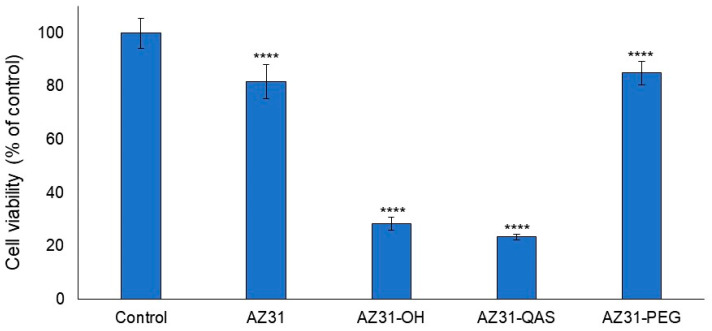
Cytotoxicity evaluation. Cell viability was measured by MTT assay in human gingival fibroblasts non-exposed (control) or exposed to the magnesium alloy AZ31 and its different derivatives. Exposure was performed for 24 h. Data report the means of two separate experiments each performed in triplicate. Error bars represent the standard deviation of the mean. **** *p* < 0.0001.

Figure 7 shows that both the AZ31-PEG derivative and unmodified AZ31 samples induce a modest decrease in cell viability (15 and 18%, respectively), thus suggesting that both sheets are largely cytocompatible. Instead, the derivative AZ31-QAS induced a marked cytotoxicity, as shown by the decrease in cell viability (77%).

### 3.5. Adhesion and Antibiofilm Activity

**Figure 8:**
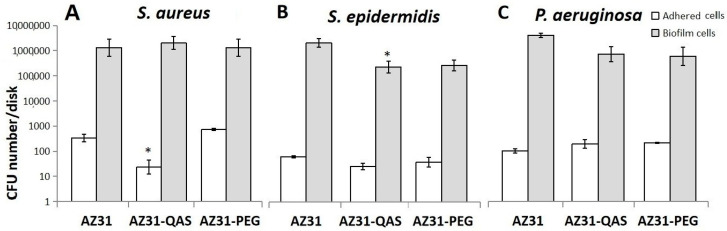
*Staphylococcus aureus* (**A**), *Staphylococcus epidemidis* (**B**), and *Pseudomonas aeruginosa* (**C**) adhesion and biofilm formation on AZ31, AZ31-QAS, and AZ31-PEF. Data are expressed as number of CFU of bacterial cells adhered to disks. Histograms represent means and standard deviation of three different determinations. * *p* < 0.05 (number of adhered cells or number of bacteria embedded in the biofilm on AZ31-QAS or AZ31-PEG versus AZ31).

The ability of different bacteria to adhere to disks and to produce a biofilm was evaluated after 90 min of contact. The adhesion of *S. aureus* to AZ31-QAS is reduced with respect to AZ31; no effect was observed for the AZ31-PEG composite. The number of bacteria embedded in the biofilm observed after 24 h of incubation was found to be similar for the three composites (Figure 8A). As regards *S. epidermidis*, the number of bacteria adherents to the different disks did not show significant differences but biofilm formation on AZ31-QAS was significantly reduced (approximately 90%) while on AZ31-PEG, a non-significant reduction in cells in the biofilm was observed (Figure 8B). No significant differences were found for the adhesion and biofilm formation of *P. aeruginosa* (Figure 8C), although the number of bacteria in the biofilm on AZ31-QAS and AZ31-PEG was lower than that observed for AZ31.

## 4. Conclusions

The aim of this study was to functionalize a magnesium alloy sheet and to evaluate the effects of the functionalization as concerns degradability, bioactivity, cytotoxicity, and antibiofilm activity.

The functionalization of AZ31 sheets was successfully achieved through the introduction of a pegylated chain or a quaternary ammonium salt. Stability tests demonstrated enhanced corrosion resistance in comparison to the AZ31 sample, accompanied by a reduction in local alkalinization and a decrease in magnesium release. In vitro bioactivity tests revealed the presence of phosphate and calcium on the surface of all the layers, particularly for the sheet derivatized with QAS. The antibiofilm tests demonstrated that the adhesion of *Staphylococcus aureus* to AZ31-QAS was diminished in comparison to AZ31. As regards *Staphylococcus epidermidis*, the biofilm formation on AZ31-QAS was markedly reduced (approximately 90%) in contrast to AZ31. Finally, the results regarding cytotoxicity towards fibroblasts indicated that the AZ31 and AZ31-PEG-derivatized sheet resulted in no cytotoxicity.

## Data Availability

The original contributions presented in the study are included in the article/supplementary material, further inquiries can be directed to the corresponding author.

## Supplementary Materials

The following supporting information can be downloaded at: https://www.mdpi.com/article/10.3390/jfb16010022/s1, Figure S1: AZ31 after 30 days in SBF. Figure S2. SEM micrographs of AZ31-QAS after 15 days of immersion. Figure S3: AZ31-PEG after 30 days in SBF. Figure S4: AZ31-QAS after 30 days in SBF.
