# Supplementary material for: The Effect of Surface Functionalization of Magnesium Alloy on Degradability, Bioactivity, Cytotoxicity, and Antibiofilm Activity
Source: J Funct Biomater. 2025 Jan 12;16(1):22. doi: 10.3390/jfb16010022 (PMC11765666; doi:10.3390/jfb16010022)
Supplement: Supplementary file 1 [file jfb-16-00022-s001.zip › jfb-3395907-supplementary.pdf]

The Effect of Surface Functionalization of Magnesium Alloy on Degradability, Bioactivity, Cytotoxicity, and Antibiofilm Activity

Morena Nocchetti [1], Michela Piccinini [1], Donatella Pietrella [2], Cinzia Antognelli [2], Maurizio Ricci [1], Alessandro Di Michele [3], Layla Jalaoui [1] and Valeria Ambrogi [1,*]

[1] Department of Pharmaceutical Science, University of Perugia, 06123 Perugia, Italy; morena.nocchetti@unipg.it (M.N.); michela.piccinini@dottorandi.unipg.it (M.P.); maurizio.ricci@unipg.it (M.R.); layla.jalaoui@libero.it (L.J.)
[2] Department of Medicine and Surgery, University of Perugia, 06132 Perugia, Italy; donatella.pietrella@unipg.it (D.P.); cinzia.antognelli@unipg.it (C.A.)
[3] Department of Physics and Geology, University of Perugia, 06123 Perugia, Italy; alessandro.dimichele@unipg.it
* Correspondence: valeria.ambrogi@unipg.it; Tel.: +39-075-5855125

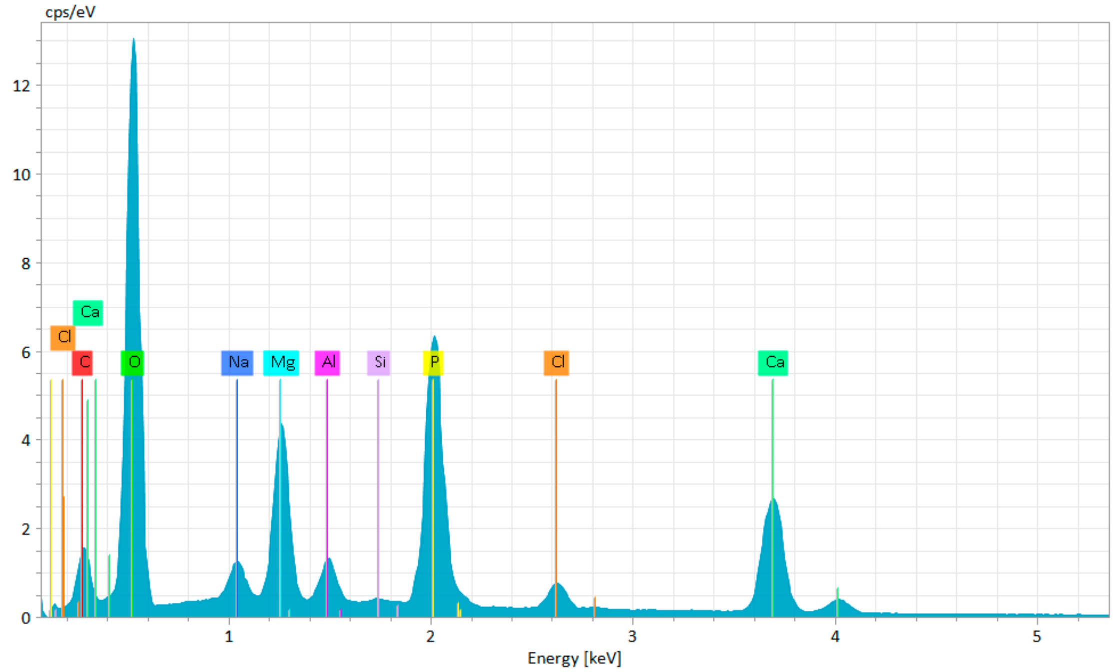

Figure S1: AZ31 after 30 days in SBF.

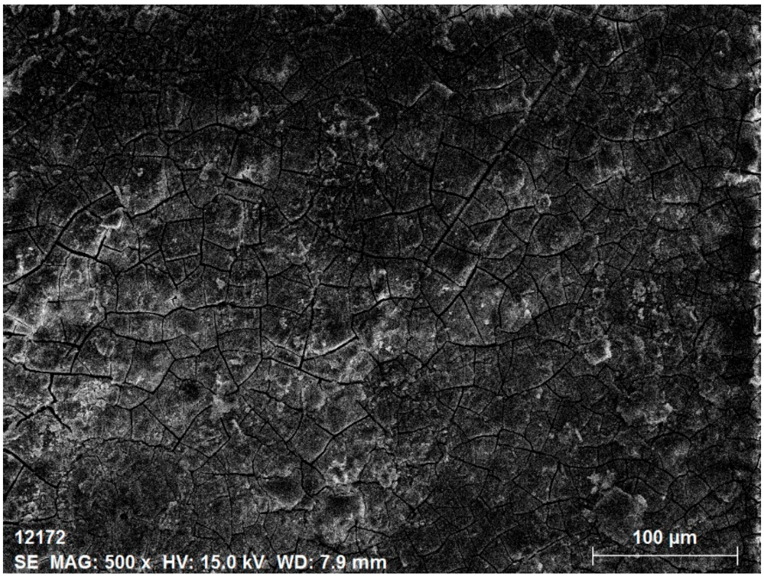

Figure S2. SEM micrographs of AZ31-QAS after 15 days of immersion.

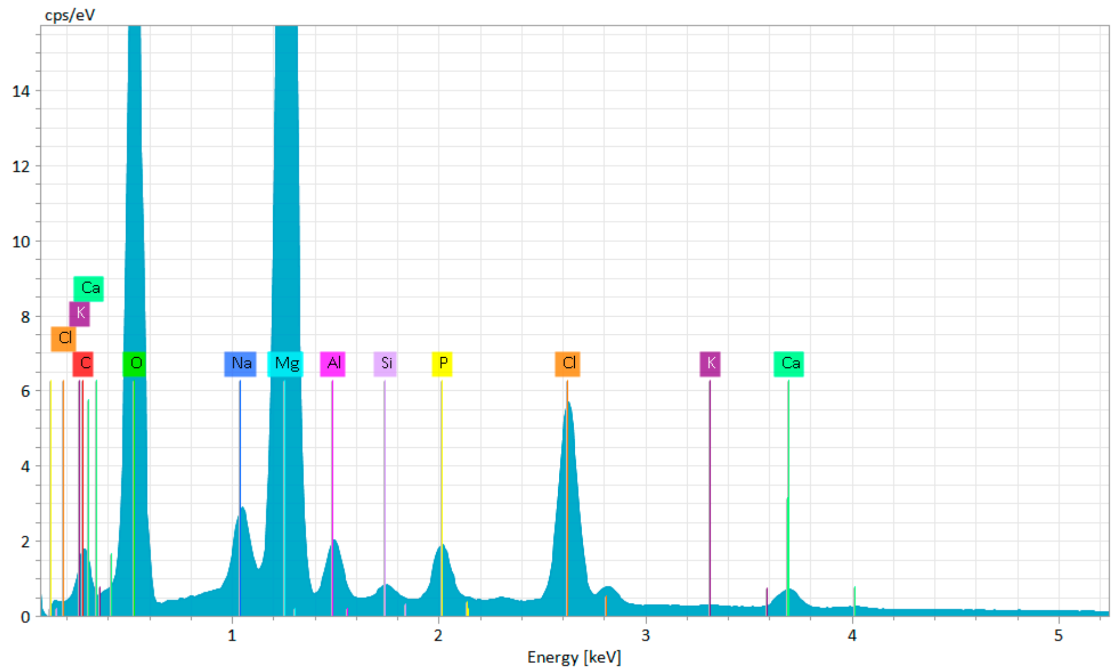

Figure S3: AZ31-PEG after 30 days in SBF.

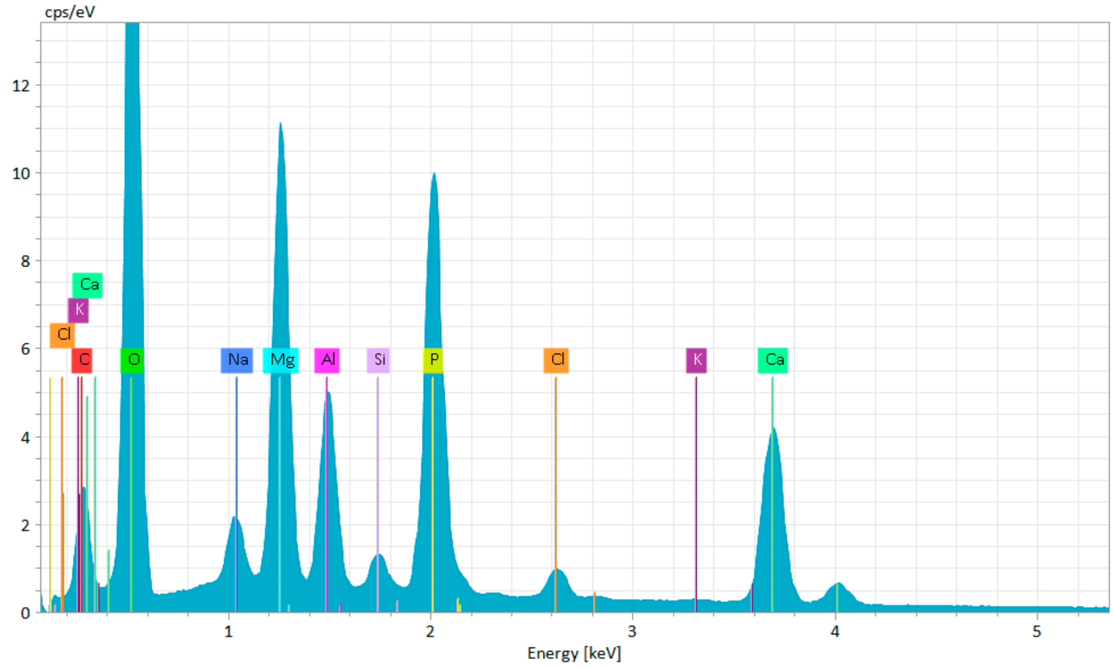

Figure S4: AZ31-QAS after 30 days in SBF.
